# Effects of blood extraction and ecophysiological experiments on stress in adult males of *Liolaemus attenboroughi*

**DOI:** 10.1242/bio.060595

**Published:** 2024-10-09

**Authors:** Fernando Duran, Marlin S. Medina, Nora R. Ibargüengoytía, Jorgelina M. Boretto

**Affiliations:** ^1^ Instituto de Investigaciones en Biodiversidad y Medioambiente, Consejo Nacional de Investigaciones Científicas y Técnicas (INIBIOMA, CONICET-Universidad Nacional del Comahue), Ecophysiology and Life History of Reptiles: Research Laboratory, Quintral 1250, 8400 San Carlos de Bariloche, Río Negro, Argentina; ^2^ Consejo Nacional de Investigaciones Científicas y Técnicas (CIEMEP-CONICET), Centro de Investigación Esquel de Montaña y Estepa Patagónica, Gral. Roca 780, 9200 Esquel, Chubut, Argentina

**Keywords:** Corticosterone, Acute stress, Liolaemidae, Haemogregarine *spp*, Lizards, Patagonia

## Abstract

Stress during laboratory experiments can affect the outcomes of ecophysiological studies. The serum corticosterone concentration (CORT), the leukocyte profile, heterophil/lymphocyte ratio (H/L), and the presence of blood endoparasites were analyzed as a proxy of stress and immunological state in adult males of the lizard *Liolaemus attenboroughi,* endemic to Patagonia, Argentina. The results of the ecophysiological variables (preferred temperature, running speed, locomotor endurance, and body condition index, BCI) were analyzed in relation to stress indicators obtained from blood samples taken at three different times: at capture, and on the third and seventh days in the laboratory. Males at capture showed a high percentage of lymphocytes and heterophils and a low of basophils, monocytes, and eosinophils. Haemogregorina-type endoparasites have been recorded in the genus *Liolaemus* for the first time. The proportion of infected males remained stable during captivity; however, these males showed higher CORT levels, increased percentages of basophils, and decreased percentages of lymphocytes. There was a significant increment in CORT and H/L, and a decrease in BCI during laboratory experiments, compared with baseline values at capture. The performance was not related to the CORT or the repeated blood sampling. The BCI decreased, possibly due to energy reserve mobilization caused by acute stress. This study shows that blood extraction and ecophysiological experiments over 7 days have a minor effect on the stress indicators used.

## Teaser

**Summary:** We study, for the first time in liolaemid lizards, the effects of blood extraction and thermal and locomotor performance experiments on stress in *Liolaemus attenboroughi* as a model species.

## INTRODUCTION

Stress can have negative effects on metabolism, vascular function, growth, reproduction, tissue repair, neuronal health, and immune defenses (Wingfield et al., 1997; Wingfield and Sapolsky, 2003; McEwen and Wingfield, 2003; Patchev and Patchev, 2006; French et al., 2010; de Assis et al., 2015; Field et al., 2023). Consequently, it becomes relevant to determine, under controlled conditions, how stressful the experiments in captive animals can be, and how the change in physiological parameters can alter the results. Previous studies have examined how handling (Sparkman et al., 2014; Borgmans et al., 2018) for a short time (Tyrrell and Cree, 1998; Seddon and Klukowski, 2012; Gangloff et al., 2017; Claunch et al., 2022), or a long time in captivity (Cree et al., 2003; Jones and Bell, 2004; Sparkman et al., 2014; Moleón et al., 2018), and repetitive blood extraction (Langkilde and Shine, 2006) can cause stress in reptiles affecting their physiology and behavior.

The effects on physiological parameters of captive maintenance can change over time, and such changes are currently difficult to predict for different reptiles (e.g. Soloaga et al., 2016; Gangloff et al., 2017). However, few studies have explored how stressful the measurement of performance variables can be for a lizard in laboratory conditions. For example, the study of Langkilde and Shine (2006) analyses the corticosterone concentration of the scincid lizard, *Eulamprus heatwolei*, against various stimuli and found that testing locomotor speed was a stressful experience, according to the increase in corticosterone concentration. Individuals in captivity can show significant changes in corticosterone concentration or leukocyte profile and yet, over time, individuals may become accustomed to captivity conditions (e.g. Seddon and Klukowski, 2012; Davis and Maney, 2018).

Ecophysiological studies include thermoregulatory capacity, thermal sensitivity, locomotor performance and endurance, bite force, and immune function, and they are relevant in determining how resilient to environmental changes in the short and long term the populations and species can be (Angilletta, 2009). In addition, information on baseline serum corticosterone concentrations in species of concern can be beneficial for improving their management and can aid in identifying and evaluating stress-related changes in hormone patterns of individuals, which is particularly relevant for threatened species breeding programs (Barry et al., 2010; Holding et al., 2014). Environmental disturbances can increase stress, the parasite load, and the risk of predation, and generate, for example, a loss of body condition (Martín and López, 1999; Pérez-Tris et al., 2004). The detriment in body condition decreases the resources allocated to the immune defense against infections and parasites (Cooper et al., 1985; Smallridge and Bull, 2000), and reduces locomotor performance, which would affect the fright response from predatory threats, or the reproductive activity (Bennett and Huey, 1990; Garland and Losos, 1994; Le Galliard et al., 2004; Miles, 2004).

Blood parameter analyses are complementary studies that are valuable in assessing and monitoring the health of wild populations (Stacy et al., 2011; Campbell, 2012; Maceda-Veiga et al., 2015). Therefore, the availability of baseline parameters from wild populations that have been poorly or non-studied is essential (Valle et al., 2018). In addition, the baseline species-specific variations in hematologic variables help to identify the effects of disease, injury, stress, or environmental conditions changes (Smyth et al., 2014; Lewbart et al., 2015). The leukocyte profile and other hematological values such as hematocrit, total erythrocyte, and hemoglobin counts, as well as clear signs of pathology, such as the presence of ectoparasites, and endoparasites in the blood, are valuable as diagnostic information about the general health status of animals (Sykes and Klaphake, 2008; Stacy et al., 2011). In most organisms, a high concentration of stress hormones (glucocorticoids, such as corticosterone) alters the number of leukocytes in circulation, increasing the number of heterophils and decreasing lymphocytes (Davis et al., 2008; Silvestre, 2014). Consequently, the heterophil/lymphocyte ratio (H/L) is frequently used to indirectly measure stress (e.g. Gangloff et al., 2017; Davis and Maney, 2018; Lamar et al., 2024). Furthermore, stressful conditions have an immunosuppressive effect and can affect disease resistance. Parasites affect their hosts’ life histories and fitness (Dunlap and Schall, 1995; Hanley and Stamps, 2002). It has been shown that the ability of the immune system to deal with parasites, such as blood endoparasites, depends partly on environmental conditions and partly on the individual's health status (Onorati et al., 2017).

Blood parameter studies have been performed in some species of Liolaemid lizards (Ceballos de Bruno, 1995; Duran et al., 2019, 2023a; Duran, 2023b; Heredia et al., 2024). The genus *Liolaemus* is one of the most diverse, with 283 recognized species (Abdala et al., 2021a). It has a wide range, extending north to the Andes of Peru and south to Tierra del Fuego in Argentina and Chile (from 10°S to 54°30′S), and from sea level to 5000 m above sea level (masl; Aparicio and Ocampo, 2010). *Liolaemus* species show great adaptive plasticity in their physiological responses to a high diversity of environments and climates (Labra et al., 2009; Ibargüengoytía et al., 2010), having been proven in numerous ecophysiological performance works (Ibargüengoytía et al., 2020; Abdala et al., 2021b). In this work, we describe for the first time the baseline values in the serum corticosterone concentration (CORT), the leukocyte profile, heterophil/lymphocyte ratio (H/L), the presence of blood endoparasites as well as the body condition index (BCI) of adult males of *Liolaemus attenboroughi,* endemic to Patagonia, Argentina. We also analyze the association of stress parameters (CORT and H/L), the presence of endoparasites, repetitive blood extractions, and BCI with relevant ecophysiological variables (preferred temperature T_p_, running speed, locomotor endurance) study in laboratory conditions.

*Liolaemus attenboroughi sp. nov* (previously described as *Liolaemus kingii*; Bell, 1843) is abundant in the northwestern Patagonian steppe of Chubut Province (Argentina; Sánchez et al., 2023) and classified as ‘not threatened’ (Abdala et al., 2012; Breitman et al., 2014). Adult individuals are robust, with a mean snout-vent length (SVL) of approximately 70 mm (Sánchez et al., 2023, mean 67.87±4.52), an omnivorous diet, diurnal activity, and a viviparous mode of reproduction (Scolaro, 2005; Sánchez et al., 2023). We hypothesized for *Liolaemus attenboroughi* that: (1) the ecophysiological experiments are stressful, and we predicted that baseline CORT values and the H/L ratio from blood samples obtained in the field would be lower than in the blood samples obtained during experimental studies in laboratory; (2) the fat storage as a proxy of individual's performance (*sensu* Frauendorf et al., 2021) would be affected by stress, and we predicted a decrease in the BCI after the experimental studies; (3) the repetitive blood extraction would affect preferred temperature (T_p_), running speed, and locomotor endurance, and we predicted a different T_p_ and lower locomotor performance in those individuals under repetitive blood extractions compared with those with a single or no blood extraction; and (4) endoparasitic infection would affect the general performance of the individuals, and we predicted that individuals infected with endoparasites would show lower BCI, lower performance, and higher CORT than those individuals without endoparasites. Even though stress responses can be very variable among species and populations, we expected to obtain a basis to understand how animals respond to stress caused by ecophysiological experiments and repetitive blood extractions to highlight the importance of experimental design as an essential step for working with both wild and captive individuals to ensure robust and reliable results. In addition, by understanding stress responses, the conditions under which animals are kept can be improved ensuring their well-being and reducing unnecessary stress.

## RESULTS

### Description of leukocyte profile

**Fig. 1.**
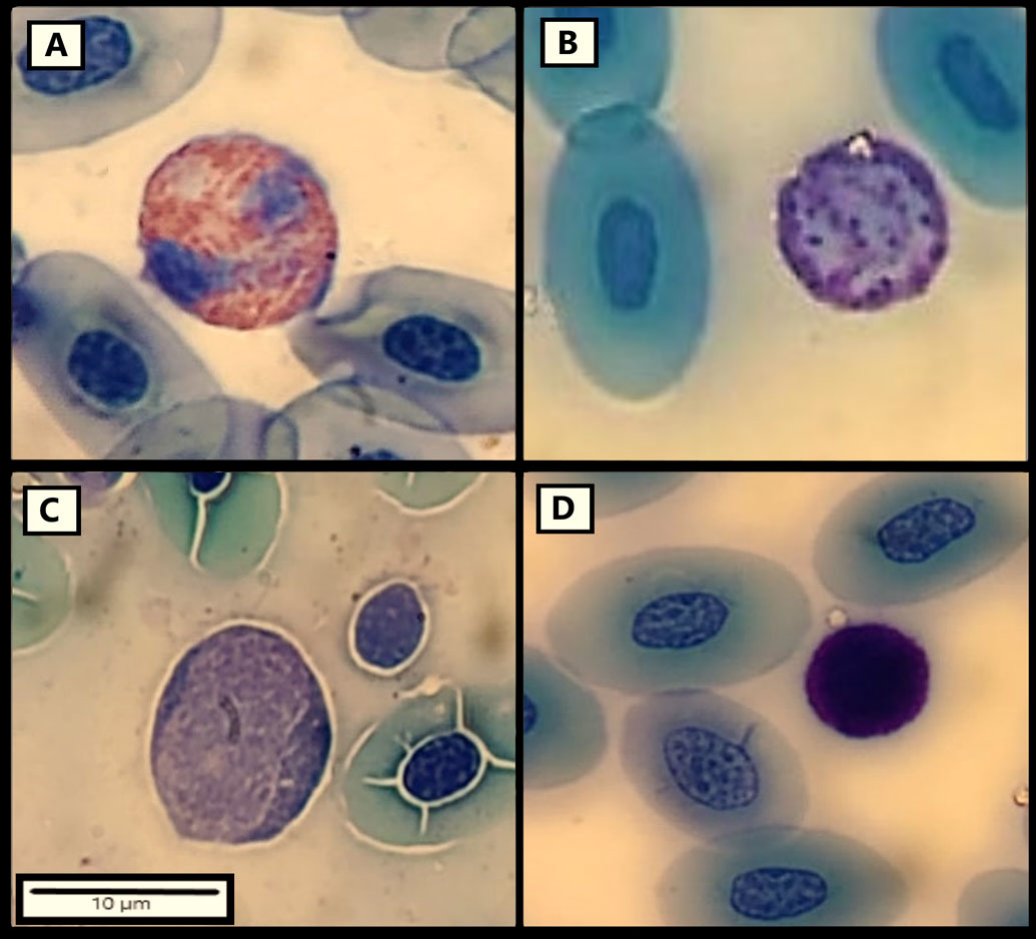
**Different types of leukocytes found in *Liolaemus attenboroughi* (*N=*19).** Heterophil (A), basophil (B), monocyte (C), and lymphocyte (D) are indicated. May-Grünwald Giemsa stain. Scale bar: 10 µm.

The most frequent cells in the leukocyte profile were lymphocytes and heterophils for the three groups of blood samples BS_1_ (at capture), BS_2_ (3 days after capture), BS_3_ (7 days after capture; Table 1). The percentages of other types of cells, such as basophils and monocytes were comparatively low, and the eosinophils were the scarcest (Table 1; Fig. 1). The heterophils showed a significant increment in BS_2_ and BS_3_ compared with the baseline values BS_1_ obtained from the same males (*N_males_*=10; paired *t-test*, BS_1_-BS_2_: *t*_9_=−3.145, *P*=0.018; BS_1_-BS_3_: *t*_9_=−3.390, *P*=0.008). Also, lymphocytes decreased in BS_3_ compared with baseline values BS_1_ (BS_1_-BS_3_: *t*_9_=2.378, *P*=0.041; Table 1). The percentage of the rest of the leukocytes did not show differences between BS_1_ versus BS_2_ and BS_3_ for the same 10 males (paired T-test or Wilcoxon signed rank test, *P*>0.05). The monocyte percentage was higher in BS_2_ than in BS_3_ when the total male sample was considered (paired T-test, *t*_18_=−2.840, *P*=0.011; *N_males_*=19), while lymphocytes, heterophils, basophils, and eosinophils percentages did not show significant differences (paired T-test or Wilcoxon signed rank test, *P*>0.05, *N_males_*=19).

**Table 1.**
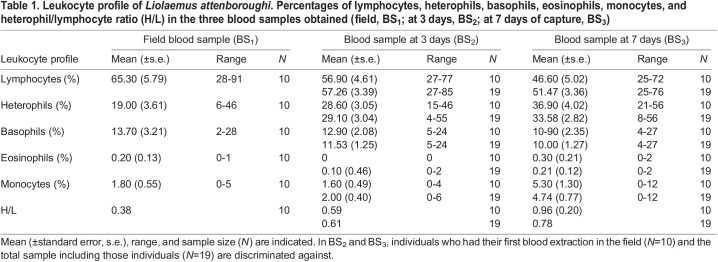
Leukocyte profile of *Liolaemus attenboroughi*. Percentages of lymphocytes, heterophils, basophils, eosinophils, monocytes, and heterophil/lymphocyte ratio (H/L) in the three blood samples obtained (field, BS_1_; at 3 days, BS_2_; at 7 days of capture, BS_3_)

**Fig. 2.**
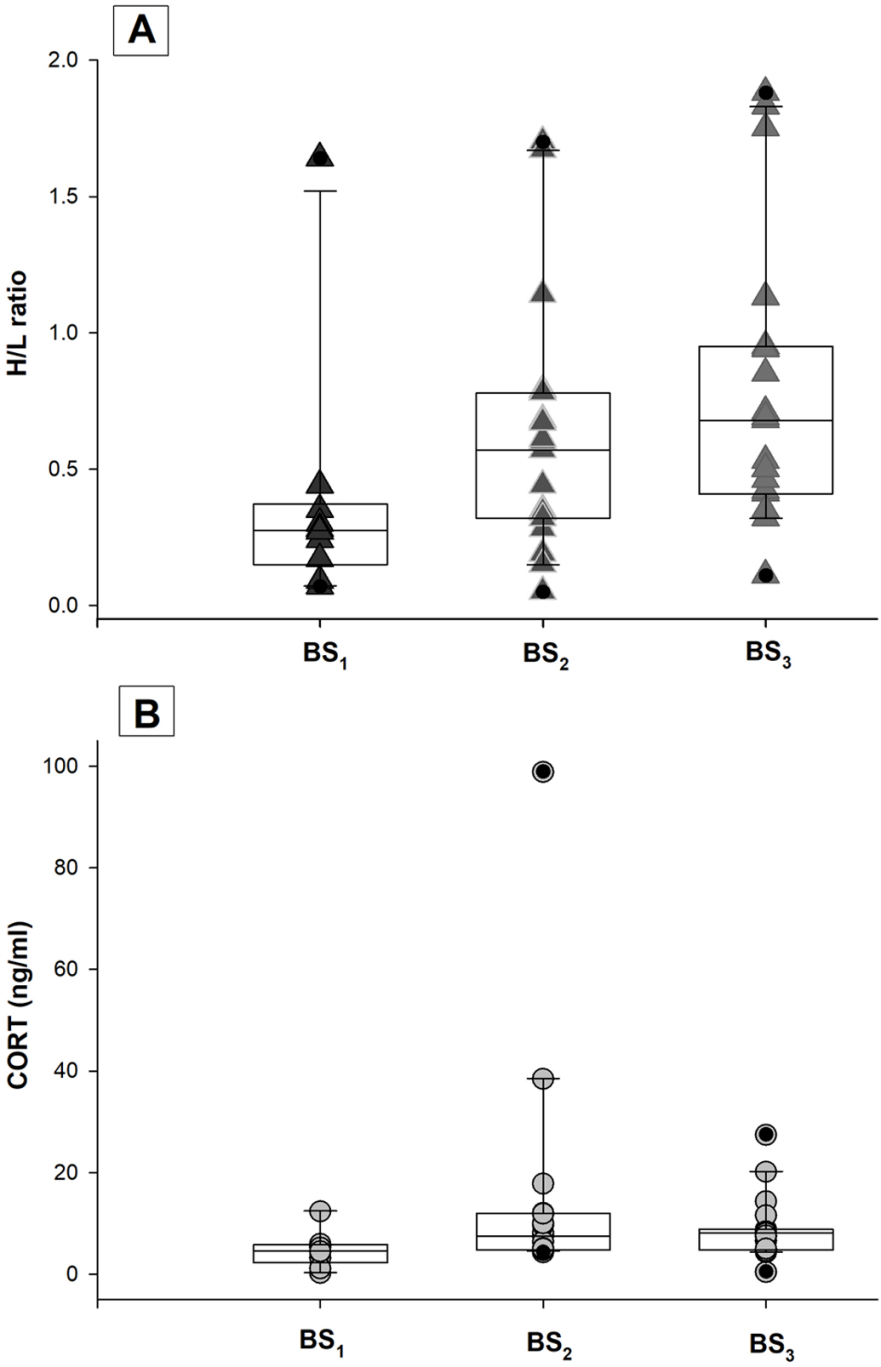
**Scatter plots representing each data, and box plots with the data**’**s quartiles, median, extreme values, and outliers.** (A) The heterophile/lymphocyte ratio (H/L ratio); (B) serum corticosterone concentration (CORT, ng/ml), obtained in blood samples drawn at the time of capture (BS_1_; *N=*10), on the third day (BS_2_; *N=*19) and the seventh day (BS_3_; *N=*19) in adult males of *Liolaemus attenboroughi*.

The H/L ratio was significantly higher in BS_2_ and BS_3_ than in BS_1_ comparing the same 10 males (BS_1_-BS_2_: paired T-test: *t*_9_=−3.289, *P*=0.009; BS_1_-BS_3_: *t*_9_=−2.350, *P*=0.043). The H/L ratio did not show differences between BS_2_ and BS_3_ for the total sample of males (*N_males_*=19; Paired *t-test*: *t*_18_=−1.292, *P*=0.106), although higher values were found in BS_3_ (Table 1; Fig. 2A). The BCI_initial_ did not show a relation with the H/L ratio of BS_1_ (simple linear regression, *P*>0.05; *N*=10), neither the BCI_final_ show a relationship with the H/L ratio of BS_3_ (simple linear regression, *P*>0.05; *N*=19).

### Serum corticosterone concentration (CORT)

Serum CORT concentration was not related to SVL of adult males in any of the three different blood samples, BS_1_, BS_2_, or BS_3_ (simple linear regression or Pearson correlation, *P*>0.05). The means of CORT concentration found in serum were: BS_1 mean_=4.75 ng/ml (range=0.27-12.38 ng/ml; *N*=10), BS_2 mean_=14.23 ng/ml (range=4.33-98.83 ng/ml; *N*=19), BS_3 mean_=8.89 ng/ml (range=0.45-27.44 ng/ml; *N*=19; Fig. 2B; Table 2). We found, for the same 10 males, a significant increment in serum CORT concentration from the baseline values BS_1_ compared with the BS_2_ and BS_3_ (BS_1_-BS_2_: Wilcoxon signed rank test, *W*=41.000; *P*=0.012; BS_1_-BS_3_: 45.000, *P*=0.004). In addition, serum CORT concentration remained high and without significant differences between BS_2_ and BS_3_ for the total sample of males (*N_males_*=19; *W*=−20.000; *P*=0.709; Fig. 2B). We found that none of the leukocytes (heterophils, lymphocytes, basophils, eosinophils, or monocytes) showed a significant correlation with serum CORT concentration of the total of males (*N*=19; Pearson's correlation, *P*>0.05); nor did serum CORT concentration correlate with the H/L ratio obtained for the total of males in BS_2_ nor BS_3_ (BS_2_, Pearson's correlation, *r=*0.027, *P*=0.911, *N*=19; BS_3_, *r=*0.398, *P*=0.091, *N*=19).

**Table 2.**
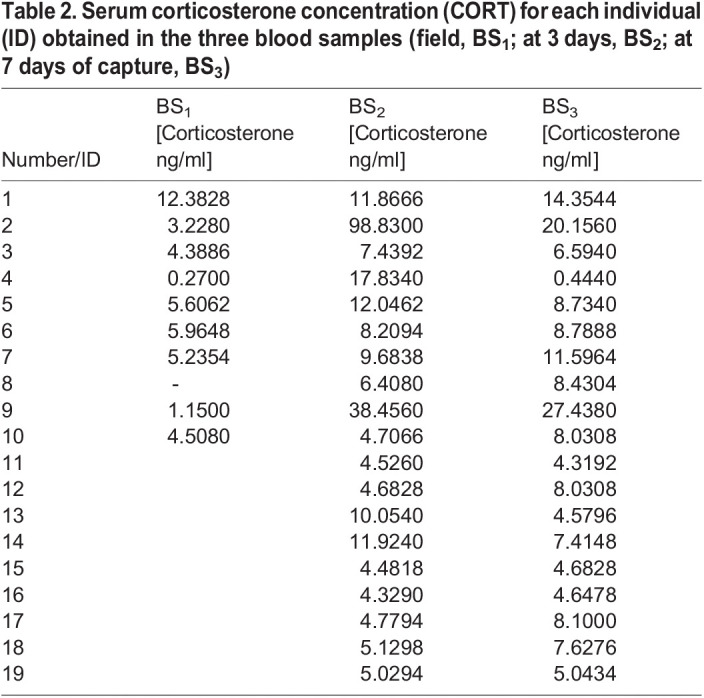
Serum corticosterone concentration (CORT) for each individual (ID) obtained in the three blood samples (field, BS_1_; at 3 days, BS_2_; at 7 days of capture, BS_3_)

The initial BCI_initial_ did not show a relation with the CORT of the BS_1_ (simple linear regression, *P*>0.05; *N*=10), neither the BCI_final_ was related with the CORT of the BS_3_ (simple linear regression, *P*>0.05; *N*=19).

### Intracellular blood parasites and their effect on ecophysiological variables

**Fig. 3.**
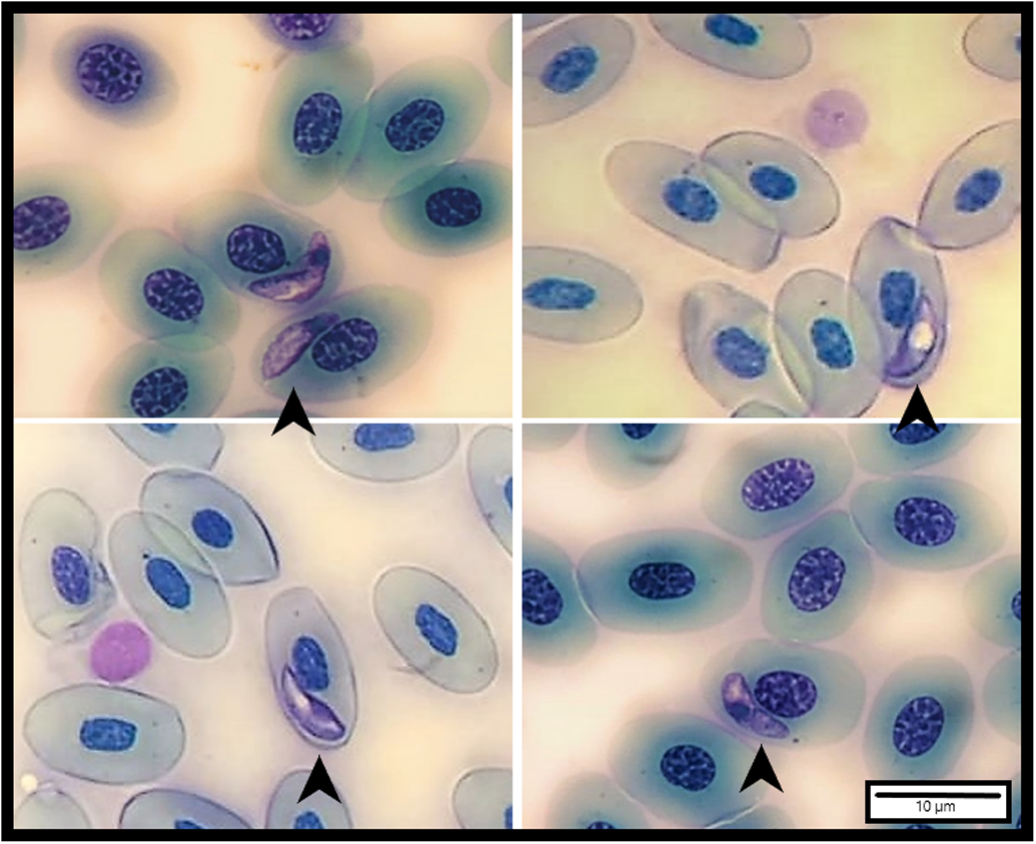
**Photomicrographs of intracellular endoparasites Haemogregarina *sp*. in adult males of *Liolaemus attenboroughi*.** May-Grünwald Giemsa stain. Scale bar: 10 µm.

We observed the presence of intracellular endoparasites in the erythrocytes of 50% of BS_1_ (*N_males_*=10). These intracellular endoparasites were identified as haemogregarines (family Haemogregorinidae, subclass Coccidiasina, Phylum Apicomplexa; Barnard and Upton, 1994; Telford, 2009; Fig. 3). Males with endoparasites in BS_1_ exhibited a higher percentage of basophils (Student's *t-test*, *t*_8_=−3.739, *P*=0.005, mean _presence_=21.4, mean _absence_=6.0), and a lower percentage of lymphocytes (*t-test*, *t*_8_=−2.742, *P*=0.025, mean _presence_=53.2, mean _absence_=77.4) than those males without endoparasites. The rest of the leukocytes and the H/L ratio did not show significant differences (*t-test*, *P*>0.05; *N*_presence_=5, *N*_absence_=5). Only basophils exhibited a significant and positive relationship with the endoparasites abundance found in BS_1_ (Pearson's correlation, *r=*0.726, *P*=0.017). Likewise, CORT concentration was higher in males with endoparasites than males without endoparasites (Student's *t-test*, *t*_7_=−2.573, *P*=0.037). On the other hand, the physiological variables measured (T_p_, SR, LR, and LE) did not exhibit a significant relationship with the abundance of endoparasites in BS_1_ (simple linear regression or Pearson's correlation, *P*>0.05).

In BS_2_, 37% of males exhibited endoparasites and a higher percentage of basophils in the leucocyte profile than males without endoparasites (Student's *t-test*, *t*_17_=−2.120, *P=*0.049, mean_presence_=14.71, mean_absence_=9.67). The rest of the leukocytes, the H/L ratio, and CORT concentration did not show significant differences between males with or without endoparasites (*t-test* or Wilcoxon signed rank test, *P*>0.05; *N*_presence_=7, *N*_absence_=12). In BS_2,_ the abundance of endoparasites did not correlate to leukocyte percentages (Pearson's correlation, *P*>0.05). The T_p_, SR, LR, and LE were unrelated to the abundance of endoparasites in BS_2_ (simple linear regression or Pearson's correlation, *P*>0.05). In addition, there were no differences in the T_p_, SR, LR, and LE between males with endoparasites (*N*=7) and those without endoparasites (*N*=12; Student's *t-test*, *P*>0.05).

The analysis of BS_3_ also evidenced that 37% of males presented endoparasites and showed a higher H/L (Student's *t-test*, *t*_17_=2.281, *P*=0.035, mean_presence_=1.11, mean_absence_=0.59) than males without endoparasites. Differences in leukocyte profile or serum CORT concentration were not found between males with and without endoparasites in BS_3_ (*t-test*, *P*>0.05). Likewise, in BS_3_, the abundance of endoparasites did not correlate with the different types of leukocytes (Pearson correlation, *P*>0.05). Neither the locomotor endurance showed a relationship with the abundance of endoparasites obtained from BS_3_ (*N*=19; simple linear regression, *P*>0.05).

### Effects of blood extraction on ecophysiological variables

We did not find significant differences in the preferred temperature (T_p_) of males that were manipulated to obtain the first blood sample in the field from those who were not exposed to blood extraction (*N_SB1_*=10 vs *N*=9_SB2_; Student's *t-test*, *t*_17_=−0.183, *P*=0.857). Similarly, differences were not found in the locomotor performance (SR, LR, and LE) between males manipulated for one (*N*=9_SB2_) or two blood extractions (*N=*10*_SB1_*) before the locomotion experiments (SR, Student's *t-test*: *t*_16_=1.321, *P*=0.205; LR, Student's *t-test*: *t*_16_=1.314, *P*=0.207; LE, Student's *t-test*, *t*_17_=0.043, *P*=0.966).

### Association between H/L, CORT, and ecophysiological variables

The SR performance showed a negative relationship with the H/L ratio obtained from the total sample of BS_2_ males (*N=*19; simple linear regression, *F*_1,17_=4.953, *P*=0.041), but the other ecophysiological variables, such as T_p_, LR, and LE did not show a relationship with the H/L ratio (simple linear regression T_p_, *F*_1,18_=1.488, *P*=0.239, *N=*19; LR, *F*_1,17_=0.499, *P*=0.490, *N=*18; LE, *F*_1,18_=0.0024, *P*=0.963). Similarly, the LE did not show any relationship with the H/L ratio obtained from BS_3_ (simple linear regression LE, *F*_1,18_=0.636, *P*=0.436).

The T_p_, SR, LR, and LE did not show a relationship with serum CORT concentration in BS_2_ (*N*=19, Pearson's correlation or simple linear regression, *P*>0.05). Similarly, the LE was not affected by serum CORT concentration from BS_3_ (*N*=19; simple linear regression, *F*_1,18_=1.839, *P*=0.193), nor by the BCI_final_ (Pearson's correlation, *r=*0.043, *P*=0.861, *N*=19). Descriptive statistics of BCI, thermophysiological variables, and locomotor performance of adult males are presented in Table 3.

**Table 3.**
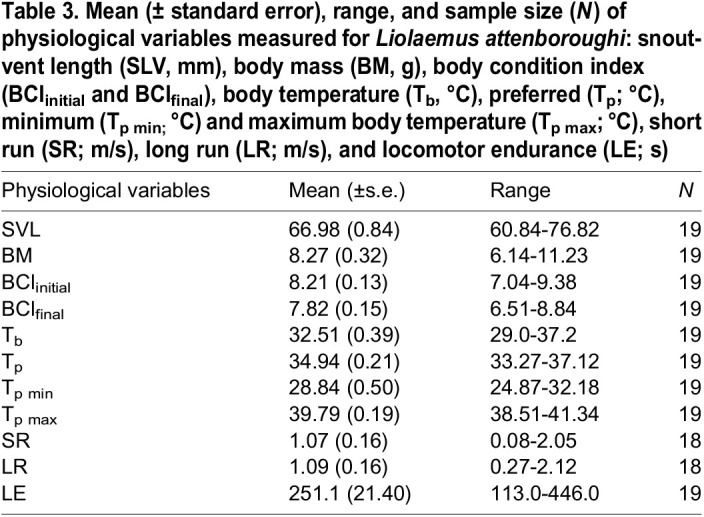
Mean (± standard error), range, and sample size (*N*) of physiological variables measured for *Liolaemus attenboroughi*: snout-vent length (SLV, mm), body mass (BM, g), body condition index (BCI_initial_ and BCI_final_), body temperature (T_b_, °C), preferred (T_p_; °C), minimum (T_p min;_ °C) and maximum body temperature (T_p max_; °C), short run (SR; m/s), long run (LR; m/s), and locomotor endurance (LE; s)

### Body condition index

We found significant differences between the BCI_inicial_ vs BCI_final_ (Wilcoxon signed rank test, *W*=−186.000; *P*<0.001, *N*=19; mean BCI_initial_=8.21; BCI_final_=7.82) since males decreased their BCI during ecophysiological experiments in laboratory conditions. However, we did not find significant differences in the BCI_initial_ or BCI_final_ between males with or without endoparasites (Student's *t*-test, *P*>0.05).

Another analysis was developed to determine if there was any relationship between the body condition index (BCI) and the thermophysiological and locomotor performance variables studied. Given that the BCI at capture (BCI_inicial_, *N=*19) was not related to any of the variables (T_p_, SR, LR, and LE; simple linear regression, *P*>0.05), it was not used as a covariate in the analyses. After all the experiments, the BCI_final_ did not show a relationship with the locomotor performance variables (SR, LR, and LE; Pearson's correlation or simple linear regression, *P*>0.05).

## DISCUSSION

### Summary of main results

Stressors cause changes in physiology and behavior, so the way that ectotherms respond to stress under laboratory conditions plays a key role in assessing results (Claunch et al., 2022). In this regard, acute stress due to blood extraction, thermoregulation, and locomotor performance experiments can be estimated using the H/L ratio and CORT concentration, resulting in good stress level indicators. We found that in adult males of *L. attenboroughi,* the H/L ratio significantly increased during ecophysiological experiments and blood extractions, compared with the values obtained in the field, immediately after capture. The H/L ratio has been a good estimator of stress levels in other lizards, such as the common wall lizard *Podarcis muralis* (Galeotti et al., 2010), the lizard *Urosaurus ornatus* (French et al., 2008), and the tuatara *Sphenodon punctatus* (Lamar et al., 2024). In addition, we found that serum CORT concentration also increased over time and maintained high values until the end of ecophysiological experiments (day 7), showing the highest levels after the third day. Although the H/L ratio and the serum CORT concentration increased due to acute stress in adult males of *L. attenboroughi*, they did not show a direct correlation among them, as has previously been observed for other ectotherms (the lizard *Plestiodon inexpectatus*, Seddon and Klukowski, 2012; the garter snake *Thamnophis elegans*, Sparkman et al., 2014; the cururu toad *Rhinella icterica*, de Assis et al., 2015). The lack of correlation could be due to the immediate changes in corticosterone concentration in blood, while changes in the H/L ratio take more time to be evidenced in blood (Jones and Bell, 2004; Sparkman et al., 2014; Gangloff et al., 2017; Davis and Maney, 2018). Blood extractions during the first hours after capture allow us to see the changes at the hormonal level, but it is possible that not show marked changes at the cellular level. Probably, repetitive blood samples in a shorter time would have allowed us to find when changes in H/L occur and if this variable correlates to CORT, but working with small lizards such as *L. attenboroughi* limits the possibility of performing repetitive extractions in short periods.

Although there is evidence of acute stress by the increments of H/L ratio and serum CORT concentrations during the experiments, thermal preference (T_p_) did not change as a consequence of blood extraction in *L. attenboroughi,* as there were no differences between males who were handled for blood extraction and those who were not. Similarly, the speeds in short and long runs, and the locomotor endurance were not affected by the number of blood extractions, suggesting that this procedure, and the acute stress it implies, do not alter physiological performance under experimental conditions. Only the speed in short runs was lower in individuals with a high H/L ratio on day three. Present results suggest that, for this liolaemid species, handling and blood extractions before carrying out ecophysiological experiments do not significantly affect the results of preferred temperature or locomotor performance. Thus, blood extraction to determine valuable hematological parameters could be used to complement ecophysiological studies. Similar results have been reported for the species *Anolis carolinensis*, which showed no effect of repeated manipulation on a series of variables taken to determine stress concentrations (Borgmans et al., 2021).

### Leukocyte profile

The baseline leukocytes profile with a high percentage of lymphocytes and heterophils and a low of basophils, monocytes, and eosinophils found in *L. attenboroughi* was similar to those described in other lizard species such as *Pogona vitticeps* (Eliman, 1997), *Leiolepis belliana rubritaeniata* (Ponsen et al., 2008), *Ctenosaura melanosterna* (Davis et al., 2011), some species of the genus *Podarcis* and *Algyroides* (Sacchi et al., 2011), and similar to *Liolaemus sarmientoi* (Duran et al., 2019; Duran, 2023b), and *Phymaturus* (Duran et al., 2023a). There was an increment in heterophils in BS_2_ and BS_3_, and in monocytes in BS_2_, and a decrease in lymphocytes in BS_3_ during ecophysiological experiments compared with baseline values obtained at capture, probably as a consequence of the acute stress experienced. Similar cellular changes attributed to acute stress due to handling and captivity conditions have also been reported for other reptiles (e.g. *Terrapene c. carolina*, Case et al., 2005; common garter snake *Thamnophis sirtalis,* Gangloff et al., 2017; *Anolis carolinensis*, Borgmans et al., 2018, *Liolaemus attenboroughi* and *Liolaemus sarmientoi,* Duran, 2023b).

### Blood endoparasite infection and its effect on thermal physiology and locomotor performance

Half of the males whose blood was drawn immediately after capture had intracellular endoparasites in the erythrocytes. These intracellular endoparasites identified as haemogregarines were previously described in lizards of the genus *Podarcis* (Amo et al., 2005; Huyghe et al., 2010; Ferreira et al., 2023), and in the lizard *Zootoca vivipara* (Oppliger et al., 1996), being the present study the first record for the family Liolaemidae. In the sample obtained immediately after capture, there was a positive relationship between endoparasites abundance and the percentage of basophils, which evidences the response capacity of this species to a parasite infection. Basophils are recognized for their association with parasitic infections and their role in modulating the innate immune response by secreting various chemicals (Stacy et al., 2011). They also participate in the inflammation process; upon activation by an antigen, they degranulate and release histamine (Campbell, 1995; Stacy et al., 2011). After 3 days of capture, all male blood samples were obtained and we found that 37% of them exhibited endoparasites infection and had a higher number of basophils in comparison with males without endoparasites. Nevertheless, this infection and the energy allocated to counteract it did not affect the physiological performance or CORT concentrations. Adult males of *L. attenboroughi* with or without endoparasitic infection selected similar temperatures under experimental conditions and exhibited similar performance in short and long runs, locomotor endurance, and serum CORT concentrations, unlike another lizard studied, *Zootoca vivipara*, where parasitized individuals showed lower locomotor speed (Oppliger et al., 1996).

### Body condition index

The BCI decreased during ecophysiological experiments and did not show any effect on the variables measured, such as preferred temperature, running speed, and locomotor endurance. Since all individuals were observed eating daily, and the presence of endoparasites did not affect the BCI, the most plausible explanation for its decrease would be an energy mobilization due to higher CORT concentrations during the 7 days, as was proposed in a previous study by Romero and Beattie (2022). This hormone is widely accepted to be responsible for the redistribution of energy reserves in the body (Romero, 2002; Moore and Jessop, 2003; French et al., 2007; Field et al., 2023).

### Final conclusions

The present study provides the first corticosterone baseline values at capture and the first results of the effects of acute stress due to capture, and manipulation during ecophysiological experiments on serum CORT concentration, immune status, and body condition in the family Liolamidae. Understanding stress responses during capture and experimental ecophysiological studies allows us to better understand their potential negative effects in wild animals. On the other hand, this study provides tools to check for acute stress when performing blood extraction to evaluate and monitor health during ecophysiological studies in the genus *Liolaemus*.

## MATERIALS AND METHODS

### Fieldwork

Field work was carried out in northwest Chubut Province, Argentina (43°S, 70°W; 630 m asl) during the activity season of lizards, in February 2020 (summer in the south hemisphere). For this study, we captured 19 adult males by hand or loop when they were active between 10:00 and 20:00 h. Immediately after capture, the body temperature (T_b_) was measured (TES 1303, ±0.03°C digital thermometer) using a thermocouple (TES TP-K01, 1.62 mm diameter) inserted approximately 5 mm inside the cloaca. A blood sample was taken from the first 10 males (BS_1_; captured between 1200 and 1500 h, considering the circadian cycle), within 5 min of capture, from the caudal vein puncture at the tail base (e.g. Amey and Whittier, 2000; Troiano, 2013; Eymann et al., 2010) with an insulin syringe (1 cc., c/a 29Gx1/2″, Terumo, not heparinized). The objective of these first 10 blood samples (BS_1_) was to provide the baseline values of the study for serum corticosterone concentration, the leukocyte profile, heterophil/lymphocyte ratio. For this, an aliquot of blood sample was immediately used to obtain a blood smear, air dried and fixed with heat, to study the leukocyte profile and the presence/absence of intracellular parasites of each individual after stained in the laboratory (Maceda-Veiga et al., 2015; described below). The rest of the blood sample (50-100 µl) was held on frozen gel packs inside a thermal bag for up to 8 h and then centrifuged twice (5 min at 3500 rpm). The serum was then collected and stored at −20°C until corticosterone assay (described below).

Males were kept in individual cloth bags until transferred to individual terraria in the laboratory. The capture site of each male was georeferenced (GPS data 3 m resolution, GARMIN) to return them to the precise capture location after completing all experiments. Captures were authorized by the Wildlife Service of the Province of Chubut (Permit #03588/16 MP; Disposition #48/08). We followed the Guidelines for the Use of Live Amphibians and Reptiles in Field and Laboratory Research of the American Society of Ichthyologists and Herpetologists (ASIH), the Herpetologists’ League (HL), and the Society for the Study of Amphibians and Reptiles (SSAR), as well as the regulations detailed in Argentinean National Law #14346.

### Laboratory conditions

All captures were made on the same day (*N=*19) to avoid differences in the time of captivity between groups of individuals. The next day, males were transferred to the laboratory located in the Centro Regional Universitario Bariloche (Universidad Nacional del Comahue), in San Carlos de Bariloche city, Río Negro Province. In the laboratory, the snout-vent length (SVL; digital caliper±0.02 mm, CA-01, Lee Tools, Guangdong, China) and body mass (BM; 100-g spring scale±0.5 g; Pesola AG, Baar, Switzerland) were recorded. Then, lizards were housed individually in open-top terraria (100×20×17 cm) supplied with a refuge, water *ad libitum*, and a 75 W incandescent lightbulb located over one end of the terrarium to supply them with heat daily from 10:00 h to 17:00 h. They remained in the same terraria in the laboratory at all times, including during the thermoregulation trial. They were fed daily with mealworm larvae (*Tenebrio molitor*) dusted with vitamins and calcium (ReptoCal, Tetrafauna™), and were observed to ensure they were feeding.

#### Body condition index (BCI)

For the estimation of *BCI*, we calculated the scaled mass index (M) of each male as an estimator of stored (fat) energy (*sensu* Peig and Green, 2009, 2010) as:

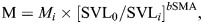
where *M_i_* and SVL*_i_* are the mass and SVL of the individual, SVL_0_ is the arithmetic mean SVL of the population, and *b*^SMA^ exponent is the standardized major axis slope from the regression of ln mass on ln SVL for the population (Peig and Green, 2009, 2010). The *b*^SMA^ exponent was calculated using the package ‘lmodel2’ (Legendre, 2015) in R (R Core Team, 2015). The body condition index for each male was calculated at the beginning (BCI_initial_) and at the end of the experiments (BCI_final_) after seven days of capture.

#### Preferred body temperature (T_p_)

The T_p_ was measured 2 days after capture, during the activity hours of the species. The thermoregulation test was carried out in the same terrarium where the males were kept (removing the shelter from the terrarium, so as not to affect the temperature selection, and with the same light and temperature gradients). The terrarium was provided with a thermal gradient (17-45°C) for thermoregulation using a 75 W incandescent lightbulb placed at the end of each terrarium. In all males, the body temperatures (T_b_) were measured every 10 s for 2 h with a temperature data acquisition module (USB-TC08, OMEGA), using an ultra-thin (0.08 mm) catheter thermocouple fastened to the belly with a hypoallergenic tape (to allow free movement of the individuals). Mean T_p_, maximum (T_p max_), and minimum preferred temperature (T_p min_), were calculated for each lizard (*N*_males_=19) following the methodology of Ibargüengoytía et al. (2010).

To analyze the effects of repetitive blood extractions on ecophysiological experiments, a blood sample was taken the day after the thermoregulation trials from all males (BS_2_; *N=*19). For some of these males (*N*=9) this was the first blood extraction, while for others (*N*=10) this blood extraction represented the second extraction (since the first, BS_1_, was obtained after capture in the field). The blood extractions (BS_2_) were performed in the same time slot as the first extraction in the field (BS_1_, 12:00 h to 15:00 h).

#### Locomotor performance

Lizards were maintained for 30 min at the mean T_p_ (34.93±0.31°C; Duran, 2023b) before the experiments of sprint and long runs (SR and LR respectively), and locomotor endurance (LE). The T_b_ was measured using a thermocouple inserted approximately 5 mm inside the cloaca connected to a digital thermometer (TES TP-K01 and TES 1303, ±0.03°C, respectively). In all experiments (SR, LR, and LE) lizards were encouraged to run by touching them gently on their hind legs or tail.

Sprint speed trials were performed on the fourth day after capture, on a racetrack 0.075 m wide and 1.20 m long, leading to a shelter. Photocells, positioned at 0.15 m intervals along the track, signaled passing lizards to a laptop that calculated speed over each 0.15 m section. Two types of runs were considered in the analyses: (1) the sprint runs (SR), considered as the speed reached between the first and the second photoreceptor (0.15 m), which is relevant for predator escape and prey capture, and (2) the long runs (LR), which corresponded to the run between the first and the last photoreceptor (1.05 m), which indicated the locomotor capability of the lizard to perform activities such as foraging, territorial defense, and courtship. Males ran three consecutive times and only the maximum run speed (V_max_) for SR and LR of the three repetitions for each individual was considered for the analyses.

Locomotor endurance trials were performed on the sixth day after capture, on a 0.5 km/h treadmill following the methodology of Sinervo et al. (2000). Endurance was defined as the time spent running on the treadmill before exhaustion, indicated by the inability of individuals to right themselves when placed on their back (Sinervo and Huey, 1990; Sinervo et al., 2000).

Once all the experiments were completed (day seven after capture from 12:00 to 15:00 h), a blood sample was taken from all males (BS_3_; *N=*19). For some of these males (*N*=9) this was the second blood extraction, while for others (*N*=10) this blood extraction represented the third extraction. This BS_3_ was also used to analyze the effects of repetitive blood extractions on ecophysiological experiments.

#### Blood samples analysis

After each blood extraction, an aliquot of blood sample was immediately used to obtain a blood smear. The remaining blood sample was centrifuged, and the serum was stored at −20°C until the corticosterone assay (described below).

The smears obtained from the three blood samples (BS_1_, BS_2_, and BS_3_) were stained in the laboratory with May-Grünwald Giemsa (Biopack^®^), which highlights the granulations and improves the staining of the erythrocytes (Martínez-Silvestre et al., 2011). The smears were used to determine the leukocyte profile (expressed as a percentage of each type of white blood cell, with a total count of 100 leukocytes for each smear, following the methodology described in Duran et al., 2019), and to determine the presence or absence of intracellular blood endoparasites. Also, during the leucocyte count the number of blood endoparasites observed was registered as a qualitative estimation of parasitic state. Leukocytes were classified using the categories established for reptiles (Stacy et al., 2011) and previously observed in other Liolaemidae species (Duran et al., 2019; Duran, 2023b), as heterophils, eosinophils, basophils, lymphocytes, and monocytes. The H/L ratio was also calculated. All slides were examined by a single observer (FD) under an optic microscope (Olympus^®^ BX51, America Inc., Melville, NY, USA; 1000X with immersion oil) equipped with a camera (TUCSEN^®^ DigiRetina16; 16mp CMOS sensor).

To determine the serum concentration of corticosterone (CORT) we used an enzyme immunoassay (EIA), following Palme and Möstl (1997). First, each serum sample was exposed to ethyl ether in a 1:10 v/v ratio, then shaken for 10 s and centrifuged for 15 min at 2600 ***g***. The supernatant was evaporated in a thermostatted bath at 40°C. It is then reconstituted in EIA buffer solution (1:10 dilution; Palme and Möstl, 1997) to perform the EIA analysis. An Ivdiagnostik M201 microplate reader was used, and each sample was analyzed in duplicate, with the coefficient of variation being less than 10%.

#### Statistical analyses

We used the statistical software programs Sigma Plot 14.0^®^ and R (R Core Team, 2015, 2021). Assumptions of normality and homogeneity of variance were tested with the Shapiro–Wilk's test and with the Levene test, respectively. When the assumptions of normality and/or homogeneity of variance were not met, we used the corresponding non-parametric test. Means are given with±1 standard error (s.e.).

A Paired *t-*test or Wilcoxon signed-rank test was used to compare, between the first and second, and the second and third blood extraction, the percentages of lymphocytes, heterophils, basophils, eosinophils, monocytes, and H/L ratio. The relationship between the different types of leukocytes (heterophils, eosinophils, basophils, lymphocytes, and monocytes) and the abundance of intracellular endoparasites in erythrocytes was analyzed by Pearson correlations. We compared, in males with or without endoparasites, the percentage of each type of leukocyte in BS_2_ using a Student's T-test and the CORT between BS_1_ vs BS_2_ and between BS_2_ vs BS_3_ using a Wilcoxon signed rank test. To determine whether CORT concentration was related to the SVL in BS_1_, BS_2_, or BS_3_, simple linear regression or Pearson correlation when assumptions for parametric tests were not met, was used. Simple linear regression or Pearson's correlation was used to relate the T_p_, SR, LR, and LE with serum CORT concentration. We also used a Student's *t*-test to compare the T_p_ and locomotor performance (short and long runs, and endurance) among individuals who had only one blood sample and individuals from whom two blood samples were drawn. R (R Core Team, 2015) was used to obtain the body condition index (BCI). Simple linear regression or Pearson's correlation was used to relate the BCI with the T_p_ and locomotor performance variables recorded.
